# The Interplay of Inflammatory Processes and Cognition in Alcohol Use Disorders—A Systematic Review

**DOI:** 10.3389/fpsyt.2019.00632

**Published:** 2019-09-12

**Authors:** Violette Coppens, Manuel Morrens, Marianne Destoop, Geert Dom

**Affiliations:** ^1^Faculty of Medicine and Health Sciences, Collaborative Antwerp Psychiatric Research Institute (CAPRI), University of Antwerp, Antwerp, Belgium; ^2^Scientific Initiative of Neuropsychiatric and Psychopharmacological Studies (SINAPS), University Department of Psychiatry, Duffel, Belgium; ^3^Department of Addiction, Psychiatric Hospital Multiversum, Boechout, Belgium

**Keywords:** inflammation, alcohol use disorder, cognition, alcohol addiction, psychiatry

## Abstract

**Rationale:** Of late, evidence emerges that the pathophysiology of psychiatric diseases and their affiliated symptomatologies are at least partly contributable to inflammatory processes. Also in alcohol use disorders (AUD), this interaction is strongly apparent, with severely immunogenic liver cirrhosis being one of the most critical sequelae of chronic abusive drinking. This somatic immune system activation negatively impacts brain functioning, and additionally, alcohol abuse appears to have a direct detrimental effect on the brain by actively stimulating its immune cells and responses. As cognitive decline majorly contributes to AUD’s debility, it is important to know to what extent impairment of cognitive functioning is due to these (neuro-)inflammatory aberrations.

**Method:** We hereby summarize the current existing literature on the interplay between AUD, inflammation, and cognition in a systematic review according to the PRISMA-P guidelines for the systematic review.

**Main findings:** Although literature on the role of inflammation in alcohol use-related cognitive deficiency remains scarce, current findings indicate that pro-inflammatory processes indeed result in exacerbation of several domains of cognitive deterioration. Interestingly, microglia, the immune cells of the brain, appear to exert initial compensatory neuroprotective functionalities upon acute ethanol exposure while chronic alcohol intake seems to attenuate these responses and overall microglial activity.

**Conclusion:** As these results indicate inflammation to be of importance in cognitive impairment following alcohol consumption and might as such provide alternate therapeutic avenues, a considerable increase in research efforts in this domain is urgently required.

## Introduction

Alcohol is the most commonly (ab)used substance worldwide with high-risk drinking occurring in up to 30% in Western populations (1). On a global scale, the WHO estimates the prevalence of alcohol use disorder (AUD) to be as high as 16% (WHO, 2004^1^) being equally present across almost all sociodemographic classes (1) WHO, 2004). Both excessive alcohol use and AUD have a profound (public) health impact as they strongly increase morbidity and mortality through inducing cardiovascular diseases, hypertension, diabetes, liver cirrhosis, and many other somatic comorbidities (1).

AUD is associated to different cognitive deficits, such as abnormalities in working memory, attention, and executive functions like response inhibition (2). As thoroughly investigated in a meta-analysis by Stavro et al. (3), these deficits remain considerable in the first 12 months of sobriety but improve after 1 year of abstinence. Nonetheless, they impact decision making in patients and thus interfere with readiness to change drinking behavior and the ability to attain abstinence (4). Moreover, poorer cognitive functioning predicts increased relapse risk over a 12-month follow-up period (5).

The specific etiopathogenesis of these AUD-related cognitive impairments is rather complex, as it may be an inherent pre-morbid trait vulnerability, but may also result from alcohol-related brain damage (ARBD). As alcohol has major neurotoxic effects, its abuse is related to mild brain atrophy that seems mostly driven by white matter loss and changes in cortical neuronal dendritic arborization (6). ARBD can either be the consequence of a direct molecular impact of the substance on the brain and/or may result from impaired liver functioning, malnutrition (vitamin B1 deficiency), and risk-taking behavior potentially associated with head injury (7). As such, cognitive alterations in AUD patients result from central as well as peripheral abnormalities (7, 8). Nonetheless, Davies et al. (9) demonstrated in a sample of abstinent (750 days) alcohol-dependent subjects without any hepatic, neurological, or other somatic impairments that deficit in visuospatial scanning, verbal memory, and processing speed were still present. These findings suggest enduring, alcohol-induced cognitive impairments. However, it should be noted that it is very likely that these cognitive impairments can both be the result of chronic alcohol use but can also reflect pre-existing cognitive impairments underlying vulnerabilities for escalating alcohol use and subsequent development of AUD. Parsons’ (10) analysis of previous studies on the relationship between alcohol consumption and cognitive deficits showed that increase in alcohol use resulted in more pronounced cognitive impairments, suggestive of a dose–response relation. Finally and most importantly, evidence is growing that not only alcohol use but also alcohol withdrawal is a neurotoxic process. Repeated detoxifications have been associated with progressive cognitive decline and impairments (11). With regard to the underlying processes responsible for the many cognition-related dysfunctions following alcohol abuse, cerebral edema, neuronal cell loss, and dysfunction of the blood–brain barrier (BBB) have been demonstrated in the brain of deceased AUD patients (12). These physiological abnormalities have further been linked to a higher concentration of CNS ammonia, mitochondrial damage, and oxidative stress caused by increased levels of reactive oxygen species (ROS) in relevant brain regions (13–15). Recently, also neuroinflammation gains more attention as to playing a role in the above neurodetrimental effects. In the last few years, neuroinflammation has been associated to cognitive decline in several pathological situations, including old age (16, 17), Alzheimer’s disease (18), schizophrenia (De Picker et al., 2018, submitted), and bipolar disorder (Van den Ameele et al., submitted). Also in AUD patients, increasing evidence points toward an aberrantly activated immune system. Excessive production of cytokines and chemokines and altered activation of microglial cells—the immune cells of the brain—have been documented in both acute and chronic phases of AUD (7, 8).

As changes in immune system activation may result in cytotoxic effects, thereby impacting neurotransmission, neuroendocrine function, and neural plasticity (19, 20), neuroinflammation presumably also at least partly contributes to cognitive deficits linked to alcohol exposure.

These findings are supported by animal studies linking inflammation to alcohol disorders and related cognitive dysfunctioning. For instance, elevated levels of the pro-inflammatory cytokines interleukin 6 (IL-6), IL-1b, and tumor necrosis factor alfa (TNF-a) have been found in the rodent brain after chronic alcohol consumption (21, 22).

Moreover, Marshall et al. (23) demonstrated (chronic) ethanol exposure in the rat to activate microglia, the brain’s most prominent immune cells, and to induce a 26% increase in anti-inflammatory cytokine IL-10 and an even larger increase (38%) in the neurotrophic factor transforming growth factor beta (TGF-b1) up to 7 days after exposure. Finally, the cellular and molecular immune system alterations induced by ethanol consumption are primarily found in the prefrontal cortex and the hippocampus of the rats; these brain regions are known to play a substantial role in several cognitive functions like memory and executive functioning (24, 25).

With this systematic review, we document the influence of AUD-related inflammation on decreased cognitive functioning observed in AUD patients.

## Method

**Figure 1:**
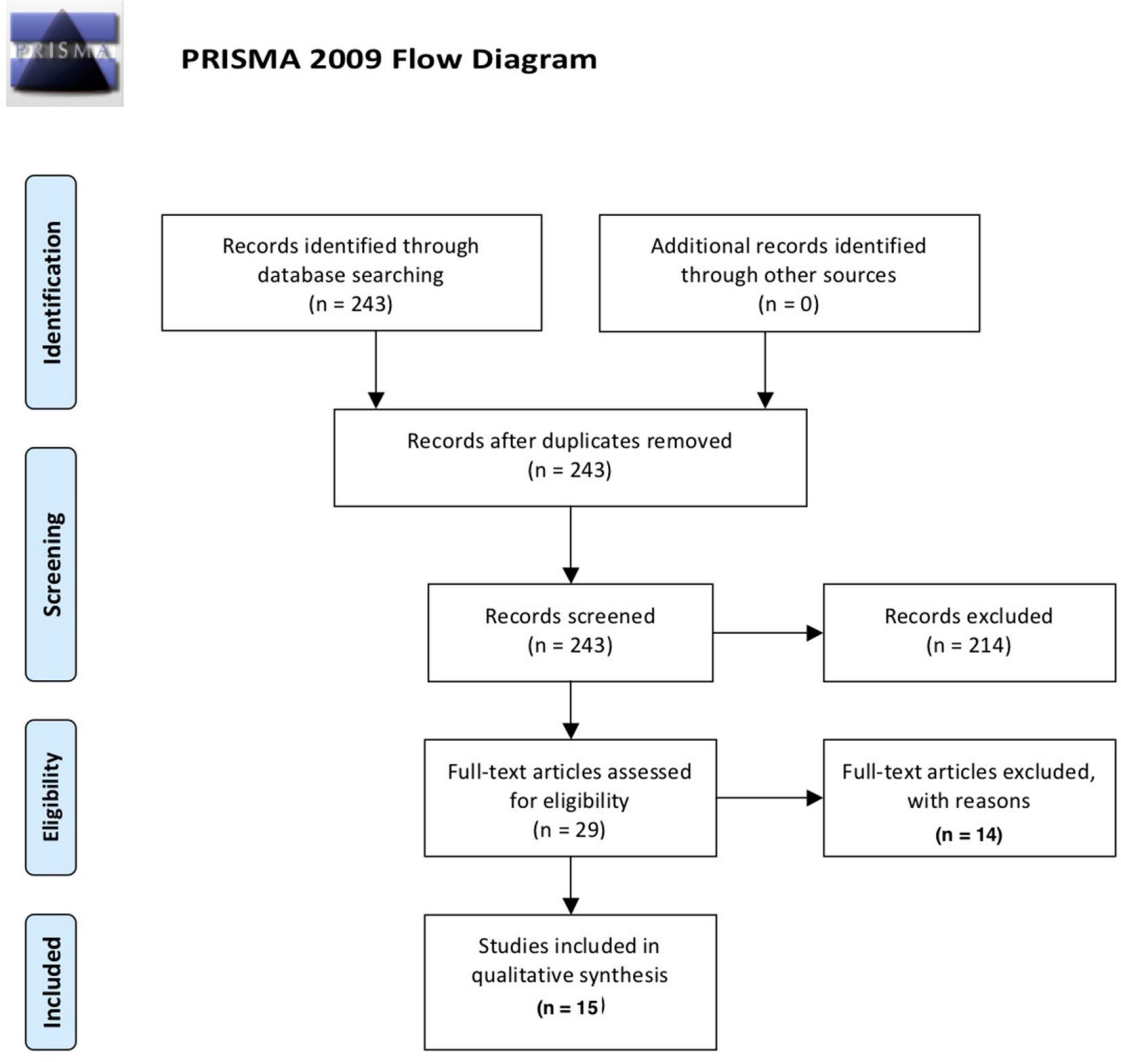
Overview of the selection process of included papers according to PRISMA guidelines (26).

This systematic review was conducted and reported according to the PRISMA-P (preferred reporting items for systematic review and meta-analysis protocols) guideline (26). We aimed to investigate all types of cognitive aberrations in individuals consuming any amount of alcohol units provided that the study discussed any type of inflammatory mediation in relation to cognition. In order to obtain original studies investigating the impact of the immune system on cognitive functioning of alcohol use and AUD, a PubMed search (January 1946–October 2018) for English language articles was conducted using the following search terms: (alcohol* OR ethyl* OR ethanol) AND (cognit* OR attention OR memory OR “executive function*”) AND (immun* OR microglia* OR inflamm* OR cytokine* OR kynuren*) NOT review[publication type] AND english[language]. The papers were filtered for human studies exclusively. An overview of the inclusion process can be found in **Figure 1**. Briefly, the above PubMed search yielded 243 results; abstract screening led to exclusion of 214 papers, leaving 29 papers that were read entirely. Of these, 16 manuscripts were deemed eligible for inclusion in the review. Of the 14 papers excluded after full-text analysis, 6 were excluded based on the absence of statistical analyses of the effect ethyl had on cognition/inflammation; 3 studies were not performed in humans; 3 studies did not concern the interaction between the immune system and cognition; 1 paper did not concern alcohol consumption; and 1 paper did not concern cognition.

Of note, the latest edition of DSM (V) refers to the disorder of being dependent on alcohol consumption as “alcohol use disorder, AUD.” However, this terminology is rather recent, and literature predating this overarching term mostly refers to this or similar conditions with “alcohol dependence” or related expressions. In order to avoid rephrasing bias in this work, we opted to retain the terminology as applied in the original paper. As such, terminology applied throughout this review remains somewhat heterogeneous in its definition of alcohol consumption disorders.

## Results

The PubMed database search resulted in an initial 243 records. Following removal of duplicates and screening of abstracts for relevance, 28 records remained. Eligibility of these 28 records was assessed by detailed evaluation of the full texts. Eight papers were deemed not relevant, and three papers concerned preclinical research, resulting in a final selection of 17 papers included in the current review.

### Relation Between the Immune System and Cognitive Functioning

Alcohol abuse has been associated with cognitive impairment in humans, mainly by deleterious effects on memory function and on executive functions including cognitive flexibility and response inhibition (27). In addition, preclinical findings link ethanol consumption to altered immune signaling and to decreased cognitive capabilities (24, 25). Also in humans, a three-way interaction between an affected immune system, alcohol use, and cognitive decline has been suggested: Miguez-Burbano et al. (28) showed that memory functioning is more severely decreased in alcohol abusing HIV-infected individuals compared to sober HIV-infected peers. Hazardous alcohol consumption was related to thymus size, and the authors suggested that the negative impact of alcohol on thymus volume was the mediating mechanism underlying impaired cognitive performance (28).

### The Association Between Alcohol Consumption, Dysregulated Cytokines, and Cognitive Functioning

In a large group of alcohol-dependent male patients (n = 78), Yen et al. (29) looked for associations between plasma cytokine concentrations and cognitive functioning. They showed that, at the start of the withdrawal period, patients display elevation of all investigated cytokines [TNF-alpha (TNF-α), interferon gamma (IFN-γ), interleukin 1 (IL-1), IL-2, IL-4, IL-5, IL-6, IL-8, IL-10, and granulocyte-macrophage colony-stimulating factor (GM-CSF)]. Although the patients displayed reduced information processing speed, these abnormalities did not correlate with cytokine levels. Nonetheless, cognitive dysfunctioning improved after 4 weeks of abstinence, which was mirrored by normalization of the cytokine levels. Felipo et al. (17) did demonstrate peripheral levels of IL-6, together with hyperammonemia, to be a driving factor for cognitive impairment, as assessed by a series of tasks addressing psychomotor and processing speed. In this line, Hanak et al. (2) recently investigated the impact of alcohol detoxification on IL-6 in a small group of patients with AUD (n = 27). They showed that when craving is caused by stress, but not when caused by alcohol or mood, IL-6 decreases after 3 weeks of detoxification. Given the small sample size in each group (n = 5) and the suboptimal statistical analysis strategies used (lack of *post hoc* exploratory analyses), these findings need to be confirmed approached with caution. The authors also aimed to find associations with cognitive functioning, and in line with the findings of Felipo et al. (17) results pointed toward more pronounced working memory deficits in the subgroup with highest levels of IL-6. Here again, methodological problems hamper interpretability. These findings thus corroborate with the previous finding that pro-inflammatory cytokines are correlated to craving in alcohol-dependent individuals (n = 52) (30). Moreover, reductions in selective attention as assessed by a self-developed validated computerized task appeared to be inversely related to the anti-inflammatory cytokine IL-10 after 3 weeks of abstinence, suggesting that reductions in protective levels of anti-inflammatory markers may lead to cognitive impairments.

Wilhelm et al. (31) investigated plasma levels of the pro-inflammatory compound tissue inhibitor of metalloproteinases (TIMP-1) and found them to be associated to self-reported memory complaints in a small sample of female (but not male) alcohol-dependent patients.

More indirect findings come from Duivis et al. (32), who demonstrated that cognitive symptoms of depression and anxiety like negative emotional state, concentration/decision making capacity, thoughts of death or suïcide,… correlate with several peripheral markers of inflammation (namely, higher levels of CRP, IL-6, and TNF-α). Interestingly, when controlling for several lifestyle factors including alcohol use, these correlations disappeared, suggesting that, at least partially, alcohol consumption mediates inflammation-associated cognitive deficiencies.

Boyer et al. (33) found a weak correlation between alcohol abuse in schizophrenic patients and cognitive dysfunctioning (mainly attention), but this was not associated to plasma CRP concentrations.

Although only a handful of studies looked into the associations between cytokines and cognitive dysfunctioning in alcohol-dependent patients, early results suggest impairments (in several cognitive domains including attention, processing speed, and memory) are associate to increases in pro-inflammatory markers and reductions in anti-inflammatory cytokines. Alcohol abstinence may partially remediate some of these deficits as mirrored by normalization of cytokines.

### The Association Between Alcohol Consumption, Altered Glial Cells, and Cognitive Dysfunctioning

Kalk et al. (34) recently investigated microglial activation *via* PET tracer imaging in newly abstinent (< 1 month) alcohol-dependent patients (n = 9). In contrast to their expectations, they found less microglial activation than healthy controls, which they suggested to be the result of a potential loss of microglia cells. Although microglia appeared to be less activated in patients, tracer binding strongly correlated with delayed verbal memory, pointing toward poor memory performance in those patients with low microglia activation. This would intuitively contrast with the report of Marshall et al. (23), who showed increased PET tracer binding/microglial activation after acute 4-day ethanol administration in rats. However, both findings can be reconciled by indications from Marshall’s study suggesting that activated microglia adopt a neuroprotective (the so-called *M2 phenotype*) rather than a neurotoxic (M1) profile in response to ethanol and that chronic alcohol abuse decreases the number of these safeguarders.

The literature investigating the impact of alcohol use and abstinence on glial activation patterns and their functionality is too scarce to be informative. However, the little preliminary data that are available suggest this to be a research avenue of interest for future studies focusing on the role of inflammation on cognitive deterioration by alcohol use.

### The Role of the Gastrointestinal System and Liver Pathologies in Inflammation and Cognitive Dysfunction in Alcohol Disorders

Many of the articles included in this review pointed toward an important role of the liver function and gut permeability and its relation with alcohol (ab) use and subsequent inflammatory processes.

Alcohol use has well known pathological effects on the liver; as such, alcoholic liver disease (ALD) is the most prevalent type of chronic liver disease worldwide. ALD is accompanied by an inflammatory presentation characterized by increases in pro-inflammatory cytokines and chemokines (35, 36). Alcohol dependence has also been associated with a leaky gut, resulting in increased permeability, which has been associated to increases in plasma lipopolysaccharides (LPS) levels, which in their turn are known to have a proinflammatory stimulating nature. These gut abnormalities and associated LPS increases normalized after a 3-week abstinence period (30). The neuroinflammation described in patients with AUD therefore results from both direct proinflammatory effects of alcohol on the brain and indirect immunological damage *via* the liver (7, 8).

Hepatic encephalopathy (HE) implies the deterioration of brain functioning arising from acute and chronic liver failure as a result of chronic alcohol abuse (7, 37). This may result in cognitive symptoms such as deficitary judgment, memory impairment, and confusion. Excess brain ammonia levels have been put forward as a strong leading factor, although *postmortem* findings suggest that a pro-inflammatory state, abnormal astrocyte, and microglial activation also seem to be involved (7, 37, 38). Dennis et al. (7) showed that HE is associated to increases in cortical IL-6 levels compared to controls or non-HE alcoholic patients, which was partly associated to microglial proliferation and activation in neuroplasticity associated brain regions (including the subventricular zone). This proinflammatory, cytotoxic environment was reflected in reduced neuronal cell counts (7). Cagnin et al. (39) investigated PK11195-binding, reflecting activated glial cells (microglia and astrocytes), in a very small sample (n = 5) of patients with HE (three of which were alcohol-induced), and found significant increases in glial activation, especially in pallidum, right putamen, and right DLPFC. Of note, the patients with the most severe cognitive impairment had the highest increases in tracer binding (including two of the alcohol-induced HE).

HE has also been shown to increase plasma IL-6 levels (40), but it should be noted that it is not clear to what extent IL-6 is able to pass the blood–brain barrier in normal physiological conditions and in HE brains (7, 41).

Furthermore, the cognitive impairments inversely related to levels of the anti-inflammatory cytokine IL-10 in AD patients as described by Leclercq et al. (30) appeared to be partly associated to increased intestinal permeability.

So, although associations between alcohol-induced immune dysregulations and cognitive dysfunctioning in the absence of liver pathology have been shown, affected liver functioning may further fuel a pro-inflammatory state peripherally and in the CNS, thereby negatively impacting cognitive functioning in AUD patients.

## Discussion

The current body of evidence suggests that acute exposure to alcohol leads to an anti-inflammatory response of the immune system (42), while chronic exposure seems to be associated more to pro-inflammatory reactions that remain present during abstinence (30). This seems to point toward an initial protective or homeostatic response of the central immune system to alcohol, whereas chronic alcohol consumption rather induces damaging pro-inflammatory states as reflected by elevation of pro-inflammatory signaling molecules (30, 43). Few studies looked into the associations between immune markers and cognitive dysfunctioning in AUD patients, and while these findings suggest that cognitive impairments (including deficits in attention, processing speed, and memory) are associated to increases in pro-inflammatory markers and reductions in anti-inflammatory cytokines, these findings are rather modest and even contradictory in nature. For example, while Cagnin et al. (38, 39) demonstrated *via* PET imaging an increase in glial cell activation in patients with HE, Kalk et al. (34) found the opposite to be true in newly abstinent alcohol-dependent patients. These discrepant findings might be allocated to the differing pathologies but might also reflect technological variability as the tracer used by Kalk et al. (34) is thought to be more specific for microglia, while that of Cagnin et al. (39) rather binds to all glial cell types in equal proportions. An overview of the findings on the interplay between inflammatory processes, ethanol exposure, and cognitive effects can be found in **Table 1**.

**Table 1:** Overview of the current literature on the interplay between inflammatory processes, ethanol exposure, and cognitive effects (NM = not mentioned in publication).

Clinical findings				
Reference	Patient population	State of ethanol exposure	Type of inflammatory response	Effect on cognition
(29)	Alcohol dependence	Early withdrawal	↑ Pro-inflammatory cytokines in peripheral blood	Reduced information processing speed
		4w abstinence	Normalization of cytokine levels	Improvement in cognitive dysfunctioning
(17)	Liver cirrhosis	NM	↑ Pro-inflammatory cytokines in peripheral blood	Reduced psychomotor and processing speed
(2)	AUD	Alcohol detoxification	↑ Pro-inflammatory cytokines in peripheral blood	Increased working memory deficits
(30)	Alcohol dependence	3-week abstinence	↑ Anti-inflammatory cytokines in peripheral blood	Reduction in selective attention
(31)	Alcohol-dependent women	Current alcohol dependence	↑ Pro-inflammatory TIMP-1	Association with memory complaints
(32)	Depression and anxiety	Alcohol use	↑ Pro-inflammatory cytokines in peripheral blood	Cognitive deficiencies
(33)	Schizophrenia	Alcohol abuse	Pro-inflammatory acute phase protein CRP	No association with attention
(34)	Alcohol dependence	Early abstinence	↓ Microglial activation	Delayed verbal memory
(39)	Cirrhosis	NM	↑ Microglial activation	More severe cognitive impairment
Post-mortem findings				
Reference	Patient population	State of ethanol exposure	Type of inflammatory response	Effect on brain
(7)	Hepatic encephalopathy (HE)	Chronic alcohol abuse	↑ Cerebral pro-inflammatory cytokines ↑ Microglial proliferation and activation	Reduced neuronal cell counts

↑, Increased; ↓, Decreased.

A hypothetical mechanistic link between neuroinflammation and cognitive decline in AUD might stem from the tryptophan catabolism (TRYCAT) pathway. This inflammatory degradation process catabolizes the essential amino acid tryptophan to kynurenine and its metabolites. It mainly occurs in microglia and astrocytes and is activated by pro-inflammatory cytokines (44). Several receptors of neuroactive kynurenine metabolites have been associated to cognitive functioning (45) (Van den Ameele et al., 2018, submitted) and might as such be of hypothetical interest to further explore the relationship between inflammation and cognitive functioning in AUD. A single study investigated the impact of retrospectively established alcohol use before, during, and after pregnancy in mothers on the cytokine and kynurenine levels in children with ADHD (mean age 10.4 +/−2.5 years), but no associations between kynurenine levels in the children on one hand and alcohol use on the other was found (46). Sadly, potential effects or correlations in the mother were not scrutinized, and no additional studies exist so far assessing the potential interaction between kynurenine and/or other TRYCAT metabolites in patients with AUD.

The relationship between immune dysregulation and cognitive deficits in AUD patients seems to be further modulated by hepatic and alcohol use–induced gastro-intestinal pathologies (38, 39), although a direct effect of alcohol on the brain will additionally contribute to this interaction. Further research should elucidate the complex interaction of alcohol use, its central and peripheral effects driving immune dysregulations, and these accumulating effects on cognitive deficits in AUD patients. Moreover, it should be investigated to what extent these effects remain after abstinence, and more importantly, to what extent immune regulatory treatment options may be protective toward cognitive functions or remediate already existing cognitive deficits in chronic users. Finally, future studies need to look into the role of potential confounding or mediating factors. First, chronic tobacco smoking has been on itself related with neuro-inflammatory processes (47). About 60% of alcohol-dependent individuals are also lifetime (heavy) smokers (48). So, the role of tobacco smoking needs to be differentiated from that of alcohol. Second, comorbidity between AUD and other mental disorders is highly prevalent. Often, these mental disorders themselves are associated with cognitive decline and neuroinflammation. For example, the prevalence of affective disorders in AUD is estimated to be 22.9% (49). Next, the most important metabolite of alcohol, acetaldehyde, has been implicated within inflammatory processes, gut permeability, and liver disease (50, 51). Alcohol metabolization is strongly influenced by genetic differences in acetaldehyde dehydrogenase activity, leaving individuals significant differences in alcohol metabolization (and acetaldehyde accumulation) between individuals and different ethnic groups. These genetic difference may represent an important aspect of the alcohol X inflammation X cognition three-way interaction.

Although we attempted to reflect on the existing literature as accurately as possible, this review contains several study- and review design–inherent limitations.

First, the DSM-V categorizes all alcohol-related disorders under the same flag of “AUD.” However, as some literature described in this review predates this novel classification, investigated populations over the included studies are often differentially defined. As such, this literature overview might encompass a heterogeneous population with differing levels of alcohol abuse as papers scrutinize “alcohol dependence” or “chronic alcohol abuse” while data were piled in this work as hallmarking the whole of AUD. Second, in addition to “classical” cognitive functions like working memory and processing speed, alcohol dependence is dependent on more complex cognitive reasonings like the sociocognitive ability to infer other’s thoughts (theory of mind). Although we did not ambition to exclude papers on the relation of inflammation with these more complex cognitions, no works were found on this topic. Likewise, although extensive literature is available on the considerable role of different domains of cognitive impairment on patient prognosis, therapeutic outcome, relapse rate,… [for review see Ref. (52)], no reports are available on the role of inflammation in AUD prognosis and outcome. As such, these relevant areas of interest remain a lacunae in this review as well. Additionally, we did not take into account potential differences in the amount of ethyl units consumed. However, we can expect the interaction between different AUD, cognition, and inflammatory parameters to be increasingly affected with increasing alcohol dosage and thus increasing inflammation.

Lastly, while extensive preclinical literature provides strong evidence for this three-way interaction between AUD, its cognitive impairments, and immune system aberrations, the actual number of studies investigating it is surprisingly low. This evidently renders making hard statements on the nature of the interactions somewhat difficult. However, this review aimed at providing sufficient indications that addressing this research avenue more in depth might vastly elucidate inflammatory pathways to be of importance in cognitive deficits marking AUD.

## Author Contributions

VC and MM performed literature search and wrote the article. MD and GD acted as advisory board on included papers and reviewed the manuscript.

## Conflict of Interest Statement

The authors declare that the research was conducted in the absence of any commercial or financial relationships that could be construed as a potential conflict of interest.

## Footnotes

1
http://www.who.int/gho/substance_abuse/burden/alcohol_prevalence/en/
